# From the Hip to the Kidney: Suspected Infection-Associated Immunoglobulin A Vasculitis in the Setting of Chronic Methicillin-Sensitive Staphylococcus aureus Prosthetic Joint Infection

**DOI:** 10.7759/cureus.109968

**Published:** 2026-05-31

**Authors:** Deydie Suarez Salazar, Angel M Green, Daniel Daugherty, David Joseph

**Affiliations:** 1 Department of Internal Medicine, East Tennessee State University Quillen College of Medicine, Johnson City, USA; 2 Department of Nephrology, James H. Quillen VA Medical Center/Mountain Home VA Medical Center, Mountain Home, USA

**Keywords:** adult-onset vasculitis, crescentic glomerulonephritis, henoch-schönlein purpura, iga vasculitis, methicillin-sensitive staphylococcus aureus, prosthetic joint infection

## Abstract

Immunoglobulin A vasculitis (IgAV), formerly known as Henoch-Schönlein purpura, is a small‑vessel vasculitis characterized by immunoglobulin A-predominant immune complex deposition in vessel walls and the glomerular mesangium. Although most commonly observed in children, adult‑onset disease is less frequent and is associated with a higher risk of significant renal involvement and poorer clinical outcomes.

We report the case of a 56‑year‑old man with alcoholic cirrhosis and chronic methicillin‑sensitive *Staphylococcus aureus *prosthetic joint infection who presented with progressive anasarca and a diffuse non‑blanching petechial rash. Laboratory evaluation demonstrated elevated inflammatory markers and acute kidney injury with a serum creatinine of 1.9 mg/dL (baseline approximately 1.0 mg/dL one month prior). Urinalysis revealed 3+ proteinuria and microscopic hematuria. Serologic testing, including antinuclear antibodies, antineutrophil cytoplasmic antibodies, anti‑glomerular basement membrane antibodies, complement levels, cryoglobulins, hepatitis panel, human immunodeficiency virus testing, and rheumatoid factor, was negative or non‑reactive. Renal biopsy demonstrated immunoglobulin A-dominant glomerulonephritis with mesangial hypercellularity and crescent formation. Skin biopsy demonstrated leukocytoclastic vasculitis. Culture obtained from the prosthetic hip abscess grew methicillin‑sensitive *Staphylococcus aureus*.

These findings support a diagnosis of suspected infection‑associated IgAV occurring in the setting of chronic prosthetic joint infection. This case highlights the importance of recognizing chronic infections as potential triggers of immunoglobulin A-mediated vasculitic disease in adults and emphasizes the need to differentiate systemic IgAV from infection‑associated immunoglobulin A-dominant glomerulonephritis.

## Introduction

Immunoglobulin A-associated diseases represent a spectrum of immune‑mediated disorders characterized by immunoglobulin A immune complex deposition. These conditions include immunoglobulin A nephropathy, selective immunoglobulin A deficiency, dermatitis herpetiformis, celiac disease, and immunoglobulin A vasculitis (IgAV). IgAV, formerly known as Henoch-Schönlein purpura, is an immune complex-mediated small‑vessel vasculitis characterized by immunoglobulin A1-dominant deposition in vessel walls and the glomerular mesangium [1,2].

IgAV occurs predominantly in children, accounting for approximately 90% of cases, most commonly between the ages of three and 15 years. Adult‑onset disease represents approximately 10% of cases and is associated with higher rates of renal involvement and worse long‑term outcomes [2,3]. Adult IgAV with renal involvement can follow a more aggressive clinical course and therefore requires careful differentiation from infection‑associated immunoglobulin A-dominant glomerulonephritis, as the diagnostic considerations and management strategies differ significantly [2,3].

The classic clinical tetrad of IgAV includes palpable purpura, arthralgia, abdominal pain, and renal involvement [1]. The etiology is multifactorial and may involve environmental exposures, medications, vaccines, infections, immune dysregulation, and genetic susceptibility [4].

Persistent antigenic stimulation may promote the production of galactose‑deficient immunoglobulin A1 (Gd‑IgA1) resulting in circulating immune complex formation and mesangial deposition [5,6]. Chronic infections caused by *Staphylococcus aureus*, including endocarditis and other deep‑seated infections such as prosthetic joint infections, have been associated with immunoglobulin A-mediated vasculitic processes [7]. Distinguishing systemic IgAV from infection‑associated immunoglobulin A-dominant glomerulonephritis remains clinically important because treatment strategies differ and immunosuppressive therapy may be harmful in the setting of active infection [8,9].

## Case presentation

A 56‑year‑old man presented with three weeks of progressive anasarca and a new petechial rash initially involving the ankles and wrists and subsequently spreading to the trunk. His past medical history was significant for alcoholic cirrhosis, heart failure with preserved ejection fraction, paroxysmal atrial fibrillation, type 2 diabetes mellitus, coronary artery disease, previously treated hepatitis C infection, and chronic left hip prosthetic joint infection with intermittent drainage.

On admission, vital signs were within normal limits. Physical examination revealed diffuse non‑blanching petechiae and palpable purpura involving the extremities and trunk without mucosal involvement. The abdomen was distended with ascites, and 2+ to 3+ bilateral lower‑extremity edema was present. A chronic draining sinus tract was observed over the left hip prosthesis. The patient denied arthralgia, abdominal pain, melena, hematochezia, or other gastrointestinal symptoms suggestive of systemic gastrointestinal involvement.

Laboratory evaluation demonstrated elevated inflammatory markers and acute kidney injury with a serum creatinine of 1.9 mg/dL. Baseline renal function one month prior demonstrated an estimated glomerular filtration rate greater than 90 mL/min/1.73 m² with a serum creatinine of approximately 1.0 mg/dL. Urinalysis revealed 3+ proteinuria and microscopic hematuria. There was no leukocytosis. Blood cultures remained negative, and there was no clinical or echocardiographic evidence suggestive of infective endocarditis.

Serologic testing, including antinuclear antibodies (ANA), antineutrophil cytoplasmic antibodies (ANCA), anti‑glomerular basement membrane (anti‑GBM) antibodies, complement levels, cryoglobulins, human immunodeficiency virus (HIV), and rheumatoid factor, was negative or non‑reactive. Because the patient had previously treated hepatitis C infection, anti‑hepatitis C virus antibodies were positive, while hepatitis C virus ribonucleic acid testing confirmed the absence of active infection. Initial laboratory findings are summarized in Table 1.

**Table 1:** Initial laboratory findings

Parameter	Patient value	Reference range
C-reactive protein (CRP)	37.2 mg/L	<10 mg/L
Erythrocyte sedimentation rate (ESR)	41 mm/hr	<20 mm/hr
Creatinine	1.9 mg/dL	0.7-1.3 mg/dL
Blood urea nitrogen (BUN)	36 mg/dL	7-20 mg/dL
Urinalysis: protein	3+	Negative
Urinalysis: red blood cells	Present	0-2/HPF
Antinuclear antibodies (ANA)	Negative	Negative
Antineutrophil cytoplasmic antibodies (ANCA)	Negative	Negative
Anti-glomerular basement membrane antibodies	Negative	Negative
Complement C3	141 mg/dL	90-180 mg/dL
Complement C4	26.4 mg/dL	10-40 mg/dL
Cryoglobulins	Negative	Negative
Hepatitis A antibody	Negative	Negative
Hepatitis B surface antigen	Negative	Negative
Hepatitis C antibody (anti-HCV)	Positive	Negative
Hepatitis C RNA	Negative	Negative
Human immunodeficiency virus (HIV)	Negative	Negative
Rheumatoid factor	Negative	Negative

Given the combination of acute kidney injury, hematuria, proteinuria, and vasculitic rash, a glomerular process was suspected. The differential diagnosis included IgAV, infection‑associated immunoglobulin A-dominant glomerulonephritis, antineutrophil cytoplasmic antibody-associated vasculitis, cryoglobulinemic vasculitis, and other immune complex-mediated glomerulonephritis. Nephrology recommended a renal biopsy to establish a definitive diagnosis.

**Figure 1:**
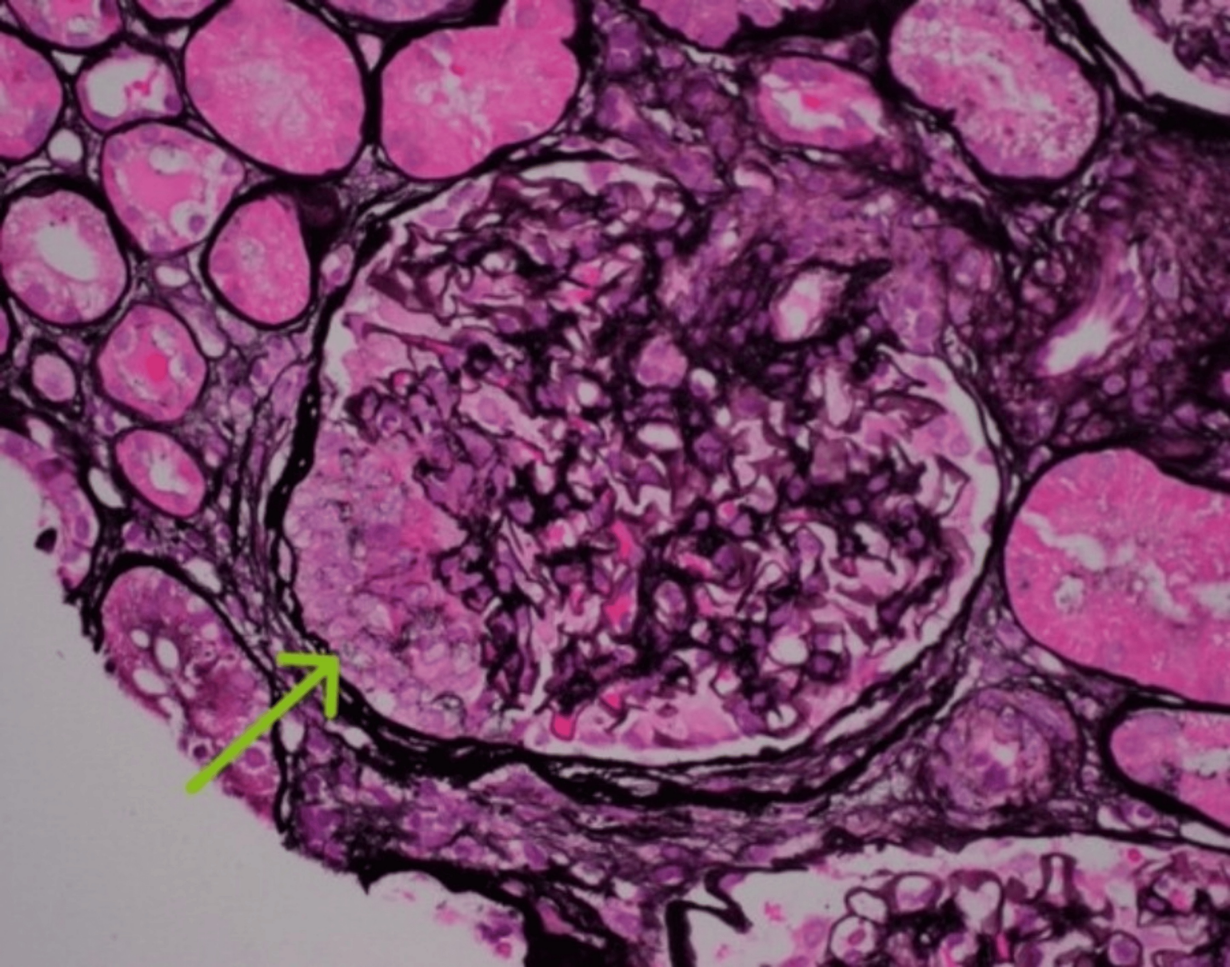
Light microscopy of the renal cortex with crescent formation Renal biopsy light microscopy demonstrating necrotizing immunoglobulin A-dominant glomerulonephritis with cellular crescent formation (arrow) and extracapillary proliferation involving the Bowman space (silver stain, approximately 400× magnification).

**Figure 2:**
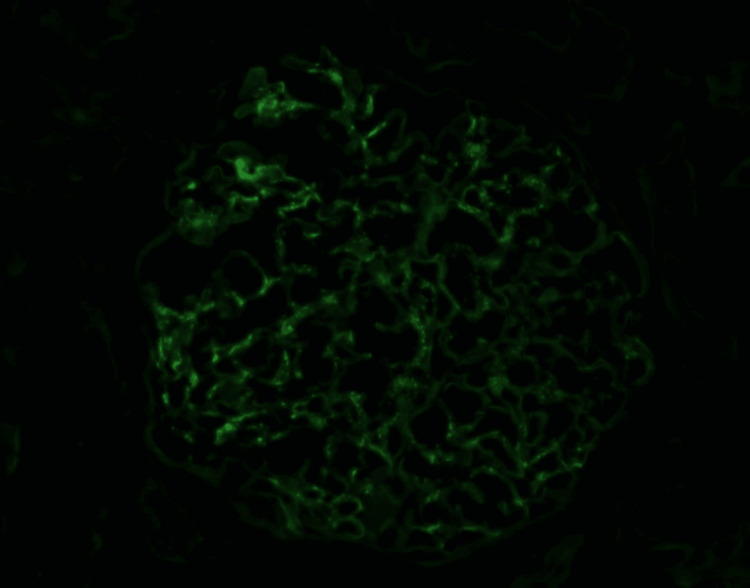
Immunofluorescence demonstrating mesangial immunoglobulin A deposition Renal biopsy immunofluorescence microscopy demonstrating dominant granular mesangial immunoglobulin A deposition within the glomerulus, supporting immunoglobulin A-mediated glomerulonephritis (immunoglobulin A immunofluorescence stain, approximately 400× magnification).

Renal biopsy demonstrated immunoglobulin A-dominant glomerulonephritis with mesangial hypercellularity, focal necrotizing features, and cellular crescent formation (Figure 1). Immunofluorescence microscopy demonstrated dominant granular mesangial immunoglobulin A deposition within the glomerulus, supporting immunoglobulin A-mediated disease (Figure 2). Skin biopsy demonstrated leukocytoclastic vasculitis involving small dermal vessels, supporting cutaneous small-vessel vasculitis. Direct immunofluorescence was not available.

Incision and drainage of the prosthetic hip abscess yielded methicillin‑sensitive *Staphylococcus aureus*. In the absence of alternative etiologies, the combination of palpable purpura, biopsy‑confirmed immunoglobulin A-dominant glomerulonephritis, and chronic methicillin‑sensitive *Staphylococcus aureus* infection supported a diagnosis of suspected infection‑associated IgAV occurring in the setting of chronic prosthetic joint infection.

The patient initially received broad-spectrum intravenous cefepime (2 g) and intravenous daptomycin (600 mg) because of concern for chronic polymicrobial prosthetic joint infection prior to definitive operative culture results. Antimicrobial therapy was subsequently transitioned to intravenous ertapenem (1 g daily) for a planned six‑week course following culture confirmation of methicillin‑sensitive *Staphylococcus aureus*. Surgical management included prosthetic hardware explantation with placement of an antibiotic spacer for infection source control.

Despite appropriate antimicrobial therapy and surgical source control, the patient's hospital course was complicated by progressive cardiopulmonary deterioration related to severe pulmonary hypertension and right ventricular dysfunction in the setting of decompensated cirrhosis and structural cardiac disease. The patient ultimately expired following transfer for advanced intervention. Renal failure was not considered the primary cause of death.

## Discussion

IgAV is defined by immunoglobulin A1-dominant immune complex deposition within small vessels and the glomerular mesangium [1]. Adult‑onset disease carries a higher risk of renal progression compared with pediatric cases [2,3].

Aberrant glycosylation of immunoglobulin A1 plays a central role in disease pathogenesis. Galactose‑deficient immunoglobulin A1 molecules form circulating immune complexes that deposit in the mesangium and small vessels, triggering complement activation and inflammatory injury [5,6]. These immune complexes may deposit in the skin, kidneys, gastrointestinal tract, and joints, producing the classic clinical manifestations of IgAV.

Chronic infections, particularly those caused by *Staphylococcus aureus*, may provide persistent antigenic stimulation that promotes immune complex formation and vascular deposition. Deep‑seated infections, including infective endocarditis and prosthetic joint infections, have been associated with immunoglobulin A-mediated vasculitic processes [7].

A key diagnostic challenge lies in distinguishing systemic IgAV from *Staphylococcus*‑associated immunoglobulin A-dominant glomerulonephritis (SAGN). SAGN typically occurs in older patients with active* Staphylococcus aureus* infection and often presents with predominant renal involvement. Hypocomplementemia may be present, and systemic vasculitic manifestations such as palpable purpura are less common. In contrast, systemic IgAV commonly presents with cutaneous vasculitis, normal complement levels, and immunoglobulin A-dominant immune complex deposition on renal biopsy [8,9].

Although direct immunofluorescence of the skin biopsy was unavailable, the patient fulfilled European League Against Rheumatism (EULAR)/Paediatric Rheumatology International Trials Organisation (PRINTO)/Paediatric Rheumatology European Society (PRES) classification criteria for IgAV based on the presence of palpable purpura and biopsy-confirmed renal immunoglobulin A deposition [10].

Management differs significantly between these entities. Treatment of SAGN primarily focuses on the eradication of the underlying infection through targeted antibiotic therapy and surgical source control when necessary. Immunosuppressive therapy is generally avoided during active infection because renal function may improve after the resolution of the infectious trigger [8,9,11].

Although *Staphylococcus aureus* infections are recognized triggers of immunoglobulin A-mediated glomerular disease, reports linking prosthetic joint infection with systemic IgAV remain limited. The novelty of this case lies in the association of chronic prosthetic joint infection with biopsy‑confirmed renal and cutaneous vasculitis and the diagnostic overlap with infection‑associated immunoglobulin A-dominant glomerulonephritis.

Limitations

As a single case report, causality between chronic infection and IgAV cannot be definitively established. Electron microscopy findings and direct immunofluorescence of the skin biopsy were not available. Additionally, long‑term renal response could not be assessed because the patient expired during hospitalization.

## Conclusions

Chronic methicillin‑sensitive* Staphylococcus aureus* prosthetic joint infection may represent a potential trigger for systemic IgAV in adults. Differentiating systemic IgAV from infection‑associated immunoglobulin A-dominant glomerulonephritis is essential because management strategies differ significantly. Recognition of infection‑associated IgAV may facilitate earlier diagnosis and appropriate management in similar clinical scenarios.
